# Cytotoxicity and oxidative stress induced by different metallic nanoparticles on human kidney cells

**DOI:** 10.1186/1743-8977-8-10

**Published:** 2011-03-03

**Authors:** Igor Pujalté, Isabelle Passagne, Brigitte Brouillaud, Mona Tréguer, Etienne Durand, Céline Ohayon-Courtès, Béatrice L'Azou

**Affiliations:** 1 Laboratoire Biologie Cellulaire, FRE CNRS 3396 Université Bordeaux Segalen, 146 rue Léo-Saignat, 33076 Bordeaux Cedex, France; 2 INSERM 1026, Bioingénierie tissulaire BioTis, Université Bordeaux Segalen, 146 rue Léo-Saignat, 33076 Bordeaux Cedex, France; 3 CNRS Université de Bordeaux, ICMCB, 87 avenue du Dr Schweitzer, 33608 Pessac Cedex, France; 4 Laboratoire Hydrologie-Environnement, UFR Sciences Pharmaceutiques, Université Bordeaux Segalen, 146 rue Léo-Saignat, 33 076 Bordeaux Cedex, France

## Abstract

**Background:**

Some manufactured nanoparticles are metal-based and have a wide variety of applications in electronic, engineering and medicine. Until now, many studies have described the potential toxicity of NPs on pulmonary target, while little attention has been paid to kidney which is considered to be a secondary target organ. The objective of this study, on human renal culture cells, was to assess the toxicity profile of metallic nanoparticles (TiO_2_, ZnO and CdS) usable in industrial production. Comparative studies were conducted, to identify whether particle properties impact cytotoxicity by altering the intracellular oxidative status.

**Results:**

Nanoparticles were first characterized by size, surface charge, dispersion and solubility. Cytotoxicity of NPs was then evaluated in IP15 (glomerular mesangial) and HK-2 (epithelial proximal) cell lines. ZnO and CdS NPs significantly increased the cell mortality, in a dose-dependent manner. Cytotoxic effects were correlated with the physicochemical properties of NPs tested and the cell type used. Analysis of reactive oxygen species and intracellular levels of reduced and oxidized glutathione revealed that particles induced stress according to their composition, size and solubility. Protein involved in oxidative stress such as NF-κb was activated with ZnO and CdS nanoparticles. Such effects were not observed with TiO_2 _nanoparticles.

**Conclusion:**

On glomerular and tubular human renal cells, ZnO and CdS nanoparticles exerted cytotoxic effects that were correlated with metal composition, particle scale and metal solubility. ROS production and oxidative stress induction clearly indicated their nephrotoxic potential.

## Background

The rapid growth of the nanotechnology industry has led to the wide-scale production and application of engineered nanoparticles (NPs). Metallic NPs are used not only in industry and medicine but also increasingly in various consumer products such as cosmetics, sunscreens, textiles and food products [1]. The success of engineered nanomaterials is due to their small size, large surface area and high reactivity. For example, a relatively inert metal or metal oxide may become a highly effective catalyst when manufactured as NPs. However, their attractive properties are also the source of reservations. *In vivo *[2-4] and *in vitro *studies [5-7] compared the toxicity of NPs with their micro-size counterparts and confirmed the higher toxic potential of NPs. The ability of NPs to induce toxicity has been attributed to their increased surface reactivity [8-10]. The smaller particles are, the more surface they have per unit mass and the more reactive they are in the cellular environment. It also has been proposed that the size of NP surface area greatly increases their ability to produce reactive oxygen species (ROS) [11,12].

NPs can enter the human body by different routes such as inhalation (respiratory tract), ingestion (gastrointestinal tract) and injection (blood circulation) [13,14]. They may then translocate to blood causing adverse biological reactions in several organs, which considered to be the secondary major sites of interaction [15-18]. The kidney is particularly susceptible to xenobiotics owing not only to its high blood supply but also its ability to concentrate toxins.

Published data indicated that numerous metallic elements are selective nephrotoxicants that preferentially accumulate and produce cellular injury in the kidneys [19,20]. Additionally, *in vivo *experiments indicated the influence of NP size or surface treatment on renal tissue distribution [21-23]. However, the impact of NPs in kidney has received little attention, yet both glomerular structures during plasma ultra-filtration and tubular epithelial cells may be exposed to NPs. Chen *et al. *[24] clearly observed damage to proximal tubular cells in mice exposed via oral gavage to copper NPs. Wang *et al. *[18] also observed signs of glomerulonephritis and pathological degeneration within the renal proximal convoluted tubules after oral administration to titanium dioxide NPs. We previously demonstrated *in vitro*, that high concentration of TiO_2 _(15 nm) NPs induced renal proximal cell death [25]. Although oxidative stress induced by NPs was suspected, the mechanisms of such effects are still unclear. The formation of ROS within cells exposed to NPs can be considered as a major contributor to their toxicological effects leading to cellular damage function.

This study aimed to screen toxicological effects of three types of NPs (TiO_2_, ZnO, and CdS) using *in vitro *cell culture models. For this purpose, the assays were conducted on two different renal cell lines: glomerular mesangial cell line (IP15) and proximal epithelial tubular cell line (HK-2). These human cell line models were used by considering two important targets in nephrotoxicity. Mesangial cells are perivascular pericytes located within the central portion of the glomerular tuft between the capillary loops and are involved in the control of glomerular hemodynamics [26-28]. HK-2 cells retain the functional characteristics of isolated proximal tubular cells and represent an alternative to primary cultures of human cells [29-31]. The three metal oxides used in this study are relevant NPs types (produced in high tonnage) and are in widespread use in a number of consumer products. TiO_2 _has excellent optical performance and electrical properties. TiO_2 _NPs are produced for applications in paints and coatings but also in cosmetics as UV-absorber. ZnO particles are widely used as polymer fillers and UV absorbers. ZnO NPs are also used as antibacterial and antifungal agents when incorporated into materials such as surface coatings (paints), textiles, and plastics. In nanotechnology, cadmium is utilized in the construction of particles known as quantum dots (QDs), which are semiconductor metalloid-crystal structures [32,33]. Owing to their small size, QDs have unique optical and electronic properties. The metabolism of QDs has not yet studied and given the fact that QD shells and coatings can be degraded, their cadmium content can be released [34].

All particles were characterized by their physicochemical properties using transmission and scanning electron microscope, turbidimetry and zetametry. For each metal form, cellular toxicity was evaluated and compared in composition and scale (nano- versus micro- particles). Finally, we identified the impact of these particles on intracellular oxidative conditions. In order to determine this impact, ROS production, glutathione content and NF-κB nuclear translocation were evaluated. The data presented here constitute the first step toward the determination of metallic NPs in renal cells and explore the role of oxidative stress in their potential toxicities.

## Results

### Nanoparticles characterization in solution

**Figure 1:**
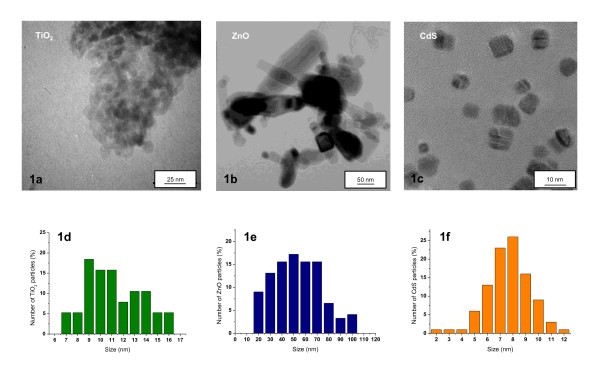
**Microscopy characterizations of NPs**. Transmission Electron Microscope (TEM) images of TiO_2 _(a), ZnO (b) and CdS (c). NPs and size distribution analysis of TiO_2 _(d), ZnO (e) and CdS (f) NPs in culture medium. (TEM scale bars (a): 25 nm, (b): 50 nm and (c): 10 nm). Images were taken at × 375K, 150K and 1000 K, respectively).

Differences in NP dispersion and agglomeration have been shown to play an important role in nanomaterial toxicology. Therefore, we determined the physicochemical properties of NPs in water and/or in culture medium. This information is not always given by the supplier, thus highlighting the importance of preliminary characterization. Detailed physicochemical characterizations of the NPs used in this study are summarized in Table 1. Size distribution was assessed using transmission electron microscopy. Figure 1 shows representative transmission electron microscopy images of TiO_2 _(Figure 1a), ZnO (Figure 1b) and CdS (Figure 1c) NPs prepared in RPMI 1640 free-serum medium. Diameter and size distribution were calculated from transmission electron microscope fitted with a camera and using ImageJ software (figure 1 - TiO_2 _(d), ZnO (e) and CdS (f)). Primary particles were uniform with a size of 7.7 ± 1.4 nm and 11.7 ± 2.2 nm for CdS and TiO_2 _NPs, respectively. ZnO NPs have a wide size distribution with an average of 75.6 ± 19 nm. These findings were in accordance with the information given by the supplier. TiO_2 _images revealed that particles tended to aggregate into larger complexes with a 38, 10 or 23-fold size increase for TiO_2_, ZnO and CdS aggregates respectively compared to the initial size of isolated particles (Table 1). Zeta potential data indicated that CdS, ZnO and TiO_2 _NPs have anionic surface charges (-14.4, -6.5 and -9.3 mV at pH 7.4 in RPMI 1640). These charges were not sufficient to stabilize suspensions via repulsive forces, leading to the creation of NP aggregates in solution. Additionally, dispersion data were performed by turbidimetry. High values were obtained with TiO_2 _and ZnO NPs in RPMI 1640 free-serum medium compared with CdS NPs. These differences are very likely due to an elevated dispersion of TiO_2 _and ZnO aggregate in medium. Additionally, in order to check that metal impurity could not be involved in cellular toxicity, ICP-OES measurements were assessed and showed no significant metal impurities (<0.01 ppm) in TiO_2_, ZnO and CdS suspensions used (data not shown). ICP-OES was also used in order to show dissolution of ZnO and CdS NPs in RPMI-1640 mediul (Additional file 1).

**Table 1:** Physical properties of investigated NPs

Particles	Source	Diameter	BET surface	Average	Agglomeration	Aggregates	Turbidimetry	Zeta potentiel	Cristal
		[nm] †	[m^2^/g] †	diameter (TEM)	state (TEM)	diameter (TEM)	in RPMI	in RPMI	structure
				[nm ± sd]		[nm ± sd]	[NTU]	[mV]	
TiO_2_	Sigma Aldrich	12 ± 2	200-220	11,68 ± 2,2	aggregate	449 ± 393	1321 ± 20 ***	-9,28	99,80%
							292 ± 3 *		anatase/rutile **‡**

ZnO	Sigma Aldrich	<100	15-25	75,61 ± 19	aggregate	722 ± 317	402 ± 32 ***	-6,45	--
							107 ± 8 *		

CdS	ICMCB	8	--	7,65 ± 1,4	aggregate	178 ± 72	11 ± 0,2 **	-14,4	--
							--		

Turbidimetry measurements were performed in RPMI 1640-serum to characterize particle dispersion rates (NTU) ± SD at 40 μg/cm^2 ^(***), 12.5 μg/cm^2 ^(**) and 10 μg/cm^2 ^(*). Average diameter of NPs, expressed as mean size ± SD nm, aggregating and average diameter of aggregates were determined using a electron microscope (TEM) fitted with a camera and Image software. (†data provided by the manufacturer, ‡data provided by [46]).

### Nanoparticles interaction with IP15 and HK-2 cells

**Figure 2:**
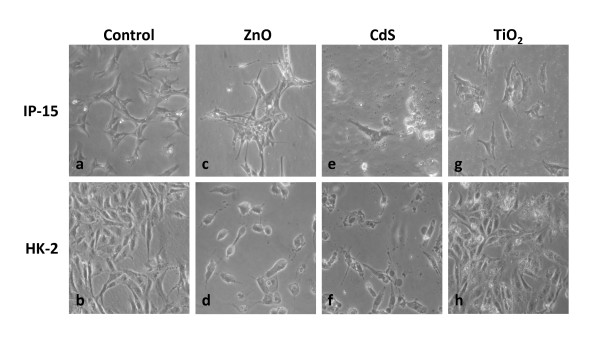
**Morphology of renal cells after NPs exposure**. IP15 and HK-2 morphology was observed using phase contrast microscope (x150). IP15 (a) cells were exposed for 24 h with different NPs: ZnO (2.5 μg/cm^2^) (c), CdS (5 μg/cm^2^) (e) and TiO_2 _(5 μg/cm^2^)(g). HK-2 cells (b) were exposed for 24 h with different NPs: ZnO (2.5 μg/cm^2^) (d), CdS (5 μg/cm^2^) (f) and TiO_2 _(5 μg/cm^2^) (h).

**Figure 3:**
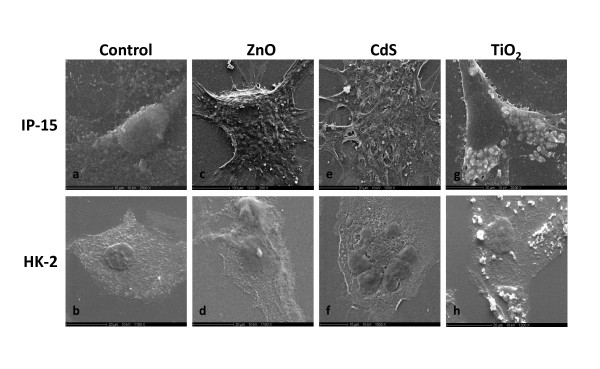
**Scanning electron microscopy observations after NPs exposure**. IP15 and HK-2 morphology was observed using Scanning Electron Microscopy (SEM). IP15 cells (a) were exposed for 24 h with different NPs: ZnO (2.5 μg/cm^2^) (c), CdS (5 μg/cm^2^) (e) and TiO_2 _(5 μg/cm^2^) (g). HK-2 cells (b) were exposed for 24 h with different NPs: ZnO (2.5 μg/cm^2^) (d), CdS (5 μg/cm^2^) (f) and TiO_2 _(5 μg/cm^2^) (h). (SEM scale bars (a, g, f): 10 μm, (b, d, e, h): 20 μm and (c): 100 μm). Images were taken at x 2500, 1000 and 200, respectively).

**Figure 4:**
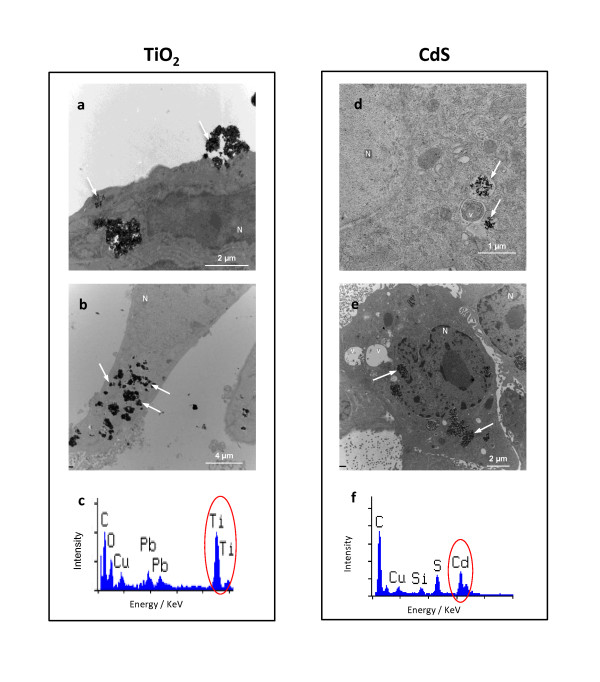
**Transmission electron microscopy observations of NPs uptake**. IP15 and HK-2 uptake and subcellular localization of NPs were observed using Transmission Electron Microscope (TEM). IP15 (a) and HK-2 (b) cells were exposed for 24 h with TiO_2 _(5 μg/cm^2^) and EDS spectra were performed in cells (c). IP15 (d) and HK-2 (e) cells were exposed for 24 h with CdS (5 μg/cm^2^) and EDS spectra were performed in cells (f) (Labels: n, nucleus; v, vesicle; arrows, NPs aggregates (TEM scale bars (a): 2 μm, (b): 4 μm, (c): 1 μm and (d): 2 μm). Images were taken at × 5K, 3.5K, 13K and 3.5K, respectively).

The effects of TiO_2_, ZnO and CdS NPs were evaluated after 24 h exposure of IP15 and HK-2 cells and were observed under optical microscopy (Figure 2). In control conditions, IP15 cells (Figure 2a) cultured in plastic dishes appeared spindle-shaped with irregular cytoplasmic projections. HK-2 cells grew continuously as a monolayer with cuboidal morphology (Figure 2b). Cytotoxic effects were visible 24 h after treatment with ZnO (2.5 μg/cm^2^) (Figure 2c and 2d) and CdS NPs (10 μg/cm^2^) (Figure 2e and 2f) in IP15 and HK-2 cells. These cells underwent morphological changes to their spherical shape, lost adhesion to the cell culture plate. No such damaged cells were observed with TiO_2 _NPs even at higher concentrations (> 20 μg/cm^2^) (Figure 2g and 2h). Scanning electron microscope photography confirmed the spherical shape of cells after 24 h ZnO and CdS NPs exposure (Figure 3c, 3d, 3e and 3f) on IP15 and HK-2 cells. Additionally, irregular nucleus shape was observed, especially with CdS NPs. No morphological change was found with TiO_2 _NPs (Figure 3g, 3h). To visualize NPs into cells, Figure 4 showed images obtained by transmission electron microscopy after 24 h NPs exposure. Selected images clearly demonstrated, for example, that TiO_2 _was taken up without affecting the cell morphology compared with the control condition (data not shown) and appeared aggregated in the vesicles in both cell types (Figure 4a and 4b, HK-2 and IP15, respectively). No NP was observed in any other subcellular compartments or the nucleus. After 24 h exposure, CdS was also observed in vesicles (Figure 4d and 4e). All particle elements were confirmed by qualitative analysis using electron dispersive x-rays (EDS) (Figure 4c, 4f).

### Cytotoxicity of nanoparticles

Effects of TiO_2_, ZnO and CdS NPs on the mortality of IP15 and HK-2 cells were assessed using 3 different assays: Neutral Red, MTT and WST-1. Cells were exposed in serum-free medium to different concentrations of NPs for 24 h. Results are expressed as percent of cell mortality compared to the control. For TiO_2 _only the WST-1 test was used because MTT and NR interacted with TiO_2 _NPs and created artifacts (data not shown). Therefore only WST-1 assay data are presented to compare TiO_2 _to ZnO and CdS NPs cytotoxicity. In relation to the cell surface dishes used (0.32 cm^2^/well), concentrations of TiO_2_, ZnO and CdS were expressed by μg/cm^2^.

#### • NP composition affects cell viability

**Figure 5:**
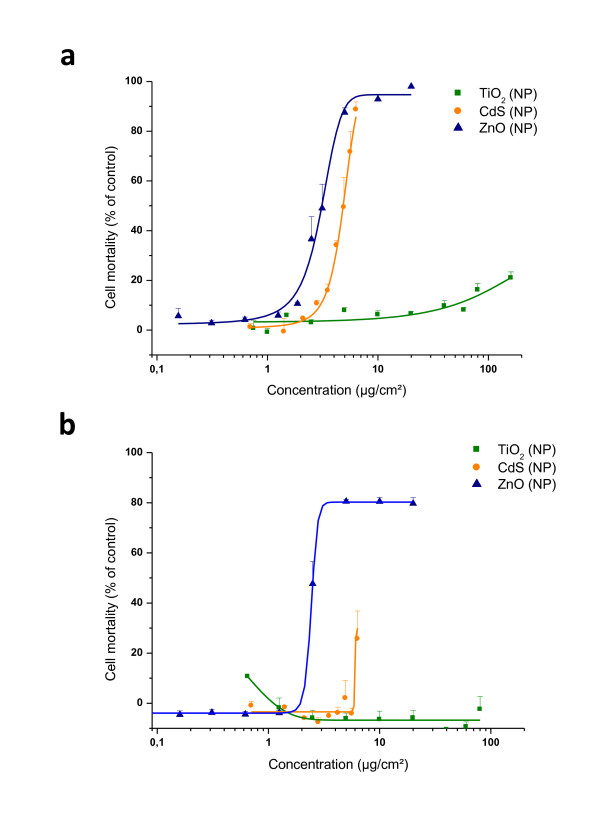
**Cytotoxicity of NPs: NP composition affects cell viability**. Effects of TiO_2_, ZnO and CdS NPs on the mortality of (a) IP15 and (b) HK-2 cells, determined using the WST-1 assay. Cells were exposed in RPMI serum-free medium with different concentrations of NPs for 24 h. Results are expressed as the percent of cell mortality compared to the control. The data are presented as the mean ± SE of at least three independent experiments.

As shown in Figure 5a, ZnO exhibited significant dose-dependent toxicity on IP15 cells. The CdS toxicity curve was found close to that of ZnO with their respective IC_50 _(3.04 ± 0. 07 μg/cm^2 ^for ZnO and 4.85 ± 0.06 μg/cm^2 ^for CdS). Results obtained with NR and MTT were similar to WST-1 (Additional file 2). For TiO_2_, only a slight toxicity was noted with 80 and 160 μg/cm^2^. As shown in Figure 5b on HK-2 cells, ZnO was again highly toxic compared to other NPs tested, with an IC_50 _calculated around 2.42 ± 0.67 μg/cm^2^. The maximal concentration used with CdS did not reach the IC_50 _but toxicity was found over 5 μg/cm^2 ^with 26 ± 2.5% of cell mortality obtained at 6.5 μg/cm^2 ^NPs. In contrast, for TiO_2 _on HK-2, no effect on mitochondrial function nor any cell mortality were observed up to 100 μg/cm^2^.

#### • NP scale affects cell viability

**Figure 6:**
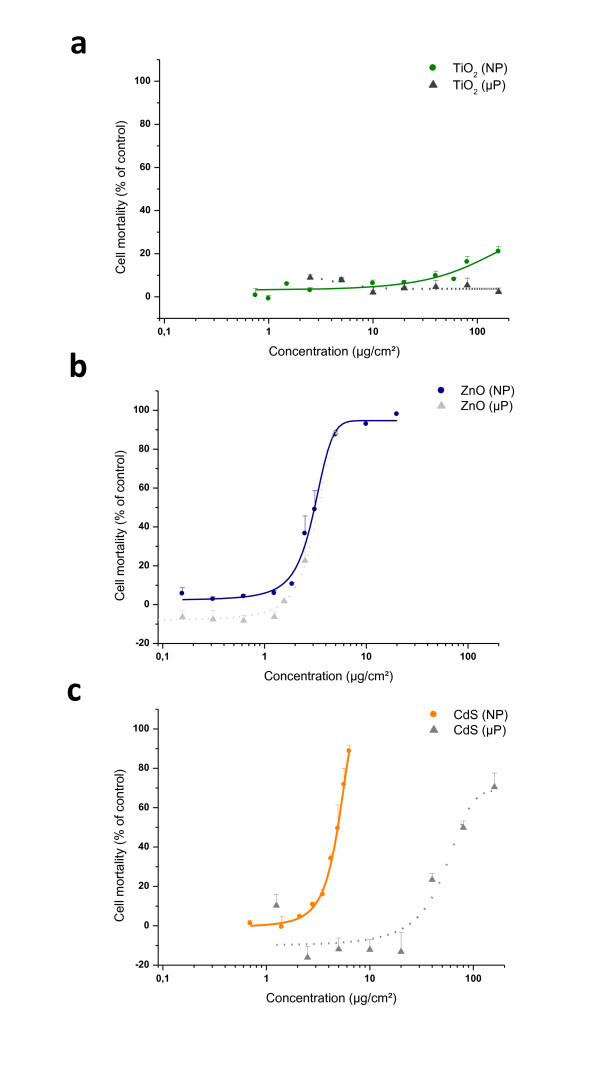
**Cytotoxicity of NPs: NP scale affects cell viability**. Comparison of NPs (<100 nm) effect on the mortality with large particles (>1 μm) of same composition, on IP15 cell type using the WST-1 assay. TiO_2 _(a), ZnO (b) and CdS (c) nanoparticles (NPs) and large microparticles (μP). Cells were exposed in RPMI serum-free medium with different concentrations for 24 h. Results are expressed as the percent of cells mortality compared to the control. The data are presented as the mean ± SE of at least three independent experiments.

Effects of NPs scale on cell toxicity were evaluated by comparing the cytotoxicity of NPs with large particles (> 1 μm). In particular, we examined IP15 cells owing to their greater sensitivity to NPs. Concerning TiO_2 _NPs (Figure 6a), no cytotoxicity was found whatever the concentration and the cell type used. ZnO induced a dose-dependent cytotoxicity with concentrations ranging from 1 to 20 μg/cm^2^. Whatever the scale used, cell mortality curves were similar with the IC_50 _calculated as 3.04 ± 0.07 μg/cm^2 ^and 3.23 ± 0.14 μg/cm^2 ^for nano- and micro-particles respectively (Figure 6b). In contrast, with CdS exposure (Figure 6c), significant differences were observed between nano- and microsized particle cytotoxicity (p > 0.05). On IP15 cells, CdS NPs exhibited more toxicity than CdS micro particles (μPs) with 16 ± 2.6% and 72 ± 8.2% cell mortality observed between 3.5 and 5.6 μg/cm^2 ^for NPs as opposed to close percentages of 24 ± 3.3% and 70 ± 7.1% obtained with higher concentrations (40 and 160 μg/cm^2^) of CdS μP. The IC_50 _were calculated to be 4.85 ± 0.06 μg/cm^2 ^for NP and 51.44 ± 7.40 μg/cm^2 ^for μP CdS respectively on IP-15 cells.

### Intracellular ROS measurement

**Figure 7:**
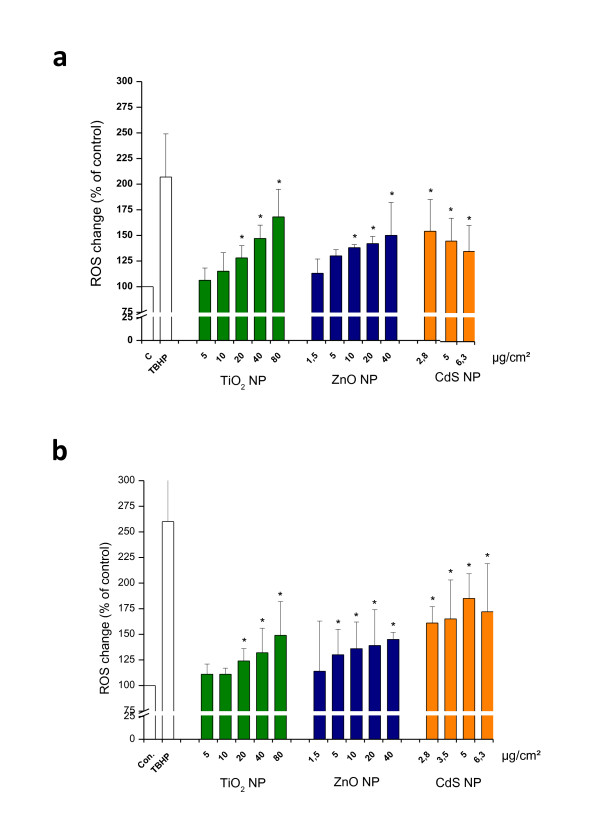
**ROS production after NPS exposure**. Effects of TiO_2_, ZnO and CdS NPs on ROS generation by IP15 (a) and HK-2 (b) cells. Cells were exposed in RPMI medium with different concentrations of NPs for 4 h, TBHP at a concentration of 50 μM were applied as positive control. The data are presented as the mean ± SE of at least three independent experiments. Data were expressed as the percentage of the ROS level in control group. Significance indicated by: *p < 0.05 versus control cells.

The ability of NPs to induce intracellular oxidant production in IP15 and HK-2 cells was assessed using DCF fluorescence, which reacts with hydroxyl radical (OH°). DCF fluorescence intensity statistically increased in a dose-dependent manner, after 4 h exposure to NPs on IP15 (Figure 7a) and on HK-2 (Figure 7b) cells compared to control (p < 0.05). The effects of the three NPs were different. CdS appeared more effective at all concentrations tested, even at low concentration (2.8 μg/cm^2^), reaching an increase of 172% fluorescence intensity (compared to control) at 6.3 μg/cm^2^. Intracellular ROS production was observed in cells treated with TiO_2 _or ZnO, with an increase of 128%, 142% for TiO_2 _and ZnO at 20 μg/cm^2 ^on IP15 and 132%, 145% for TiO_2 _and ZnO at 40 μg/cm^2 ^on HK-2.

### Intracellular oxidative stress levels

**Figure 8:**
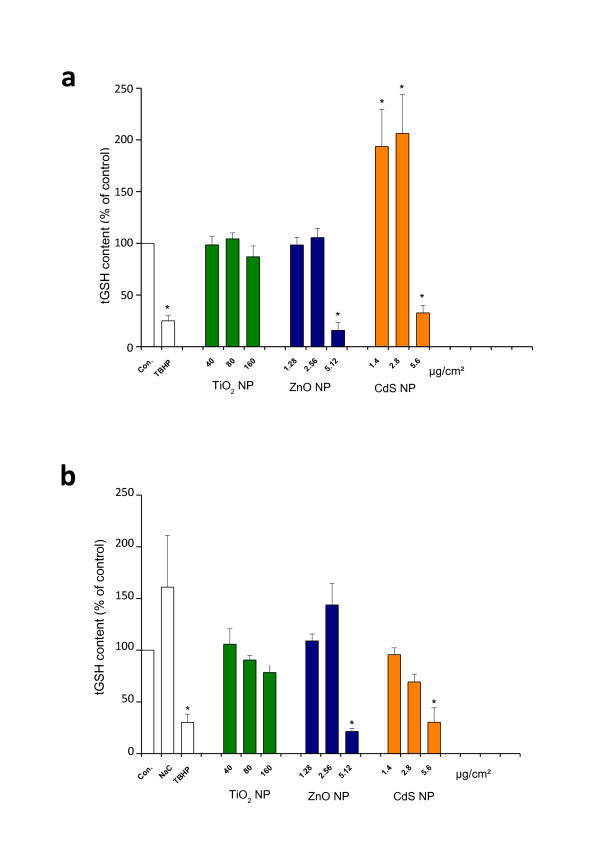
**GSH after NPS exposure**. Effect of TiO_2_, ZnO and CdS NPs on tGSH level, measured after 24 h exposure in IP15 (a) and HK-2 (b) cells. As control, TBHP at a concentration of 50 μM and NAC at a concentration of 20 μM were applied. Data were expressed as the percentage of the tGSH level in control group. Data presented mean ± SE of at least three independent experiments. Significance indicated by: *p < 0.05 versus control cells.

**Figure 9:**
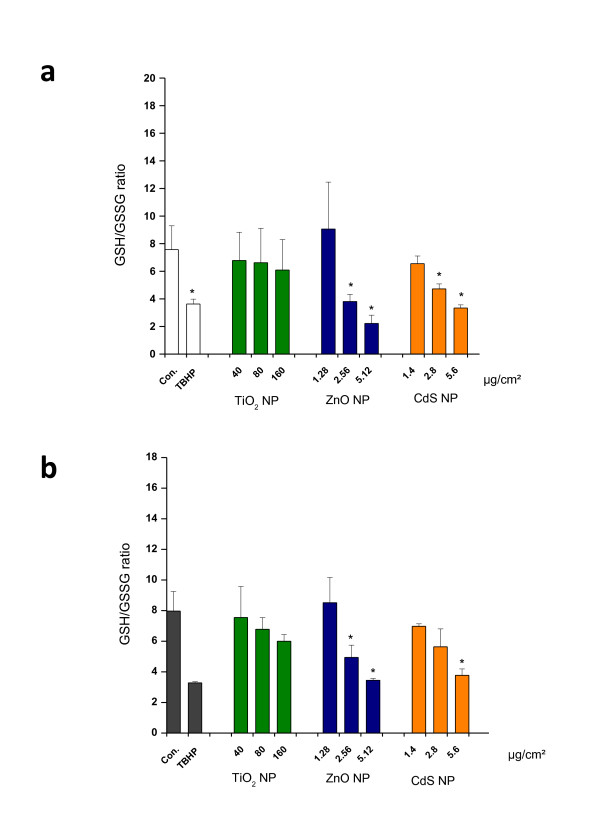
**GSH/GSSG after NPs exposure**. Effects of TiO_2_, ZnO and CdS NPs on GSH/GSSG intracellular level. IP15 (a) and HK-2 (b) cells were exposed in RPMI medium with different concentrations of NPs for 24 h. As positive control, TBHP was applied at a concentration of 50 μM. Data are expressed as the ratio of the GSH level on GSSG level. Data presented mean ± SE of at least three independent experiments. Significance indicated by: *p < 0.05 versus control cells.

Alteration in total GSH (tGSH) level content in cells can be considered as an indication of adaptive response of the cell to oxidative damage. As shown in Figure 8, both ZnO and CdS at high concentrations significantly decreased the tGSH level in IP15 (Figure 8a) and HK-2 (Figure 8b) cells compared with control values (p < 0.05). Intracellular tGSH was greatly reduced (15 ± 7% and 21 ± 3%) with 5.12 μg/cm^2 ^for ZnO, and (32 ± 7% and 30 ± 14) with 5.6 μg/cm^2 ^for CdS NPs on IP15 and HK-2 cells respectively, indicating functional damage to both cell types. Using low and non-toxic concentrations, we observed a significantly large increase in tGSH especially for CdS on IP15 cells, indicating a possible glutathione synthesis by the cells (as shown when NAC was added). For TiO_2 _NPs, no significant reduction in tGSH level was observed even at 160 μg/cm^2 ^(86 ± 10% for IP15 and 78 ± 8% for HK-2), indicating no obvious adaptive cell response with TiO_2 _NPs on both cell types. The oxidative state of the cell was also observed by determining GSH and GSSG levels, since cellular oxidative stress leads to an imbalance in GSH homeostasis. Both ZnO and CdS NPs (>2.56 μg/cm^2^) statistically decreased the GSH/GSSG ratio in IP15 (Figure 9a) and HK-2 cells (Figure 9b) compared with control values (p < 0.05), the data indicating a statistically significant oxidative stress in both cell types. For TiO_2 _NPs no significant decrease was observed up to 160 μg/cm^2 ^on both cell types indicating no obvious oxidative stress at these concentrations.

### Evidence of NF-κB nuclear translocation

**Figure 10:**
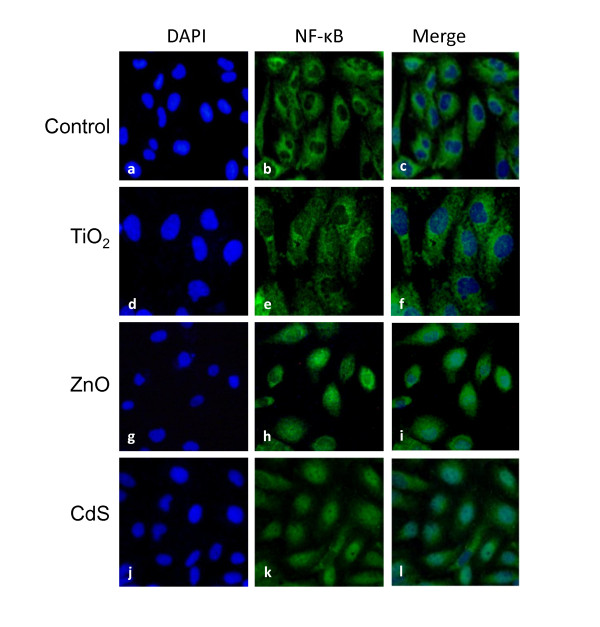
**NF-κB nuclear translocation after NPs exposure**. Effects of NPs on NF-κB nuclear translocation in HK-2 cells. Cells were exposed for 1 h with different NPs: TiO_2 _(20 μg/cm^2^), ZnO (5 μg/cm^2^) and CdS (5 μg/cm^2^). After immunostaining of DAPI (a, d, g, j) and p65 subunits of NF-kB (b, e, h, k), cells were observed under fluorescent microscopy (X200 excepted for (d), (e),(f) with X400 magnification) and representative photos are shown. Merge images were showed (c, f, I, l). Data represented mean ± SE of the fluorescence intensity on the nuclei expressed as a percentage of control (untreated) cells.

Cells were also used to investigate the nuclear transduction of NF-κB by NPs using a fluorescent staining technique. NF-κB is an important upstream regulator of various cytokines induced in response to diverse stimuli as well as ROS. Figure 10 shows fluorescence in the cells stained with an antibody to the p65 subunit of NF-κB after treatment with NPs. Nuclei were visualized using DAPI fluorescent which binds strongly to DNA (Figure 10: control (a), TiO_2 _(d), ZnO (g), CdS (j)). A dose of 20 μg/cm^2 ^for TiO_2 _and 5 μg/cm^2 ^for ZnO and CdS were chosen for this experiment to minimize interference in fluorescence observation. As shown in Figure 10 with merge images (DAPI/NF-κB), ZnO (i) and CdS (l) NPs induced a strong NF-κB nuclear translocation (81% and 46% of cells after 1 h exposure of CdS and ZnO) compared to control (22%)(c). After TiO_2 _exposure, no evident nuclear translocation was observed with only 27% of fluorescent cells after 1 h (f) and 4 h (data not shown).

## Discussion

Nanoparticles (NPs) were found to reach the systemic circulation after inhalation, ingestion or intravenous injection. They are known to disseminate to several organs such as liver, spleen, kidneys, brain or heart [13,35-37]. Kidneys play an important role in eliminating xenobiotics from the body and thus, NPs absorbed in the systemic circulation can be excreted by renal clearance [38,39]. Such translocation depends on the physicochemical properties of NPs, and their migration to distant sites is an important issue with regard to their toxicity. This study aimed to investigate human renal cell responses to manufactured NPs in order to highlight their potential toxicity and/or biological responses.

Until now, studies described the potential toxicity of NPs on pulmonary targets [13,40], while little attention has been paid to renal tubular and glomerular targets. Some *in vivo *experiments evidenced damages with morphological, pathological, and cellular changes leading to kidney dysfunction after exposure to NPs [18,24]. In a recent *in vivo *study, Kim *et al.*, [41] showed a dose-dependent accumulation of silver NPs in the glomeruli and in the basement membranes of renal tubules after inhalation or ingestion. The kidney is composed of different cell types with varying sensitivities to toxic substances. In this study, we selected two *in vitro *cell models which targeted a distinct essential portion of the kidney *i.e*. the contractile mesangial cells (IP15) from the glomeruli involved in the control of glomerular hemodynamics [27,28] and HK-2 proximal epithelial cells involved in accumulation and tubular reabsorption [29,31]. Therefore, this study is of particular interest regarding the development of routine screening tests and for investigating the precise mechanisms of action of NPs.

The NPs used (TiO_2_, ZnO and CdS) differ in their composition, size and solubility, three parameters that are highly involved in nanomaterial-induced toxicity [9]. TiO_2_, ZnO and CdS NPs were chosen for their applications as manufactured compounds. The metallic component of NPs is known to induce different responses in the human body [42,43]: cadmium is a toxic metal that can damage the kidney; zinc is an essential metal for the human organism and titanium is a harmless, non-toxic metal. By creating a large surface area, their nano-size plays an important role in their reactivity with the cellular environment by greatly increasing their ability to produce reactive oxygen species (ROS) [11,12]. Numerous physicochemical parameters are thought to be critical determinants of nanomaterial toxicity: size, crystalline structure, chemical composition, and surface area [44,45]. However, no single parameter has yet been identified as the one responsible for toxicity. Nevertheless, several physicochemical properties are important to establish before performing toxicity assays. NPs were analyzed by electron microscopy in order to evaluate the size of isolated and aggregated NPs. Additionally, turbidimetry measurements were also undertaken to characterize particle dispersion rates, while a Zetasizer was used to determine zeta potential. More details were available from other studies using the same NPs in which the physicochemical characteristics were in accordance with our data [46,47]. All measurements were performed under conditions close to toxicity experiments in the RPMI 1640 free-serum medium. NPs in media were most of the time in large aggregates compared to the initial size of isolated NPs. This aggregation was due to a low anionic charge of the particle surface, which was insufficient to stabilize suspensions via repulsive forces, thus leading to the creation of aggregates in solution. The larger aggregates (TiO_2 _and ZnO) were no longer influenced by Brownian motion and tended to sediment, unlike CdS which demonstrated colloidal dispersion.

Cytotoxicity of NPs was evaluated after 24 h using cytotoxicity assays with colorimetric tests. Several studies have shown that reagents (NR, MTT, LDH) can bind to NPs and produce invalid results due to NPs/dye interactions or adsorption of the dye/dye products [25,48]. Wörle-Knirsch *et al. *[49] showed that A549 cells incubated with carbon nanotubes (CNTs) mimic a strong cytotoxic effect of roughly 50% after 24 h in the MTT assay, whereas the same treatment with WST-1 detection revealed no cytotoxicity. In our study, interferences were also observed with TiO_2 _NPs. Abnormal absorbance increases expressed as abnormal cell viability increases were found with 40 μg/cm^2 ^TiO_2 _using Neutral Red (125%) and MTT (120%) assays probably due to the ability of this NPs to fix neutral red or formazan crystals dye. WST-1 indicator dye has the advantage of being water-soluble and stable in the culture medium, showing no alteration in cell viability. Given these interactions, cytotoxicity was compared using WST-1 data. It has frequently been suggested that metal impurities associated on NPs can contribute to their cell toxicity, especially iron, which can trigger ROS production by Fenton's reaction. In our study, no toxic metal impurities were found using ICP-OES assays, confirming that no metal impurities associated on NPs can influence the cell response.

In the literature, it has been suggested that the smallest material have the greatest toxicity. Compared with their micro counterparts, TiO_2_, and CdS induced different dose-dependent toxicity conferred by nano-scale reactivity. However, like the results observed with micro and nano ZnO particles, toxicity also varied due to other particle physicochemical properties. At the nanoscale level, the initial size of NPs was not necessarily related to their toxic potential. With close NPs size, CdS (10 nm) and TiO_2 _(11 nm) induced different toxic effects. TiO_2 _NPs of small size (11.7 nm) and greater specific surface (200-220 m^2^/g) were less toxic than Zn NPs of larger size (75.6 nm) and a lower reactive surface (15-25 m^2^/g). In the biological environment, NPs also tend to form aggregates of larger size than primary ones (>100 nm). In our study, no clear relationship could be demonstrated between aggregate size and toxicity-dependent effect; ZnO and CdS had a similar toxicity curve but aggregate size and primary size were totally different. Braydich-Stolle *and al. *[50] have already observed significant differences in toxicity with similar agglomerated NPs (1800 nm). Thus, at the nanoscale level, others parameters have been proposed as critical determinant, i.e. chemical composition, solubility, oxidation status and cell types.

On both cell types, ZnO NPs were found to be the most cytotoxic and CdS had a closer toxicity than ZnO. Toxicity can be explained in part by the release of Cd^2+ ^elements and/or size, surface and cellular uptake [51]. This result is in accordance with *in vitro *studies which highlighted NPs toxicity closely related to their solubility [52]. The release of Cd^2+ ^or Zn^2+ ^ions can induce toxicity and cellular dysfunction. This dissolution can increase under acidic conditions as well as in the presence of biological components such as amino acids and peptides. In contrast, TiO_2 _induced low toxicity probably due to its insolubility. The effects on NPs solubility was underscored in the study by Brunner *et al. *[53], where insoluble NPs (TiO_2_, CeO_2 _and ZrO_2_) induced fewer cytotoxic effects than soluble NPs (ZnO, Fe_2_O_3 _and Ca_3_(PO_4_)_2_).

Our results also indicated differences in cytotoxicity depending on cell type; both cell types showed different sensitivities depending on the NPs used. Jin *et al. *[54], showed TiO_2 _NPs toxicity at high dose (600 μg/ml) in fibroblast cells (80% of viability) during 24 h exposure, whereas Wang *et al. *[18] found the same toxicity in lymphoblast cells at medium dose (65 μg/ml). The impact of cell type in the cytotoxic responses could be due to differences in cell function. Owing to the physiological role of epithelial tubular cells by active and passive cell transport of metabolism products and xenobiotics, these cells are involved in intensive accumulation [55]. Human HK-2 tubular epithelial cells appear to be less sensitive to NPs than other tubular cells as LLC-PK_1 _and less sensitive than glomerular cells. Based on our results, it appears that the chemical composition and solubility of NPs are not only major determinant in cellular responses. But, these effects may be also accentuated or attenuated according to the cell type and tissues exposed.

In the second part of this study, we investigated the ability of our NPs to cause cytotoxicity by altering intracellular oxidative conditions in ROS production. The generation of ROS by NPs is generally considered to a major contributor to NPs toxicity and their formation, by exceeding the cellular defensive capacity and causing oxidative damage to biomolecules. ROS can also affect cell function by directly acting on cell components, including lipids, proteins, and DNA, by destroying their structure, ultimately leading to cell death. In this study, intracellular ROS production was measured through the production of 2',7' dichlorofluorescein (DCF) using fluorescence assay. DCFH-DA revealed intracellular production of hydroxyl radical (OH°). Oxidative stress can be expressed in terms of glutathione (GSH) and glutathione disulfide (GSSG) ratio in the cell. GSH is an essential antioxidant that is oxidized during oxidative stress to form a GSSG disulfide [56]. Our results demonstrate that NPs induced ROS in a dose-dependent manner, by surface reactivity of particles and/or by release of soluble metal compound, leading directly or indirectly to ROS production. This suggests that ROS production initiated by NPs induces different cellular responses, depending on the chemical properties of particles. Soluble NPs, like CdS and ZnO, induce the production of intracellular ROS, which leads to an imbalance in antioxidant defense. Cells attempt to restore this imbalance by activation of NF-κB transcription factor, which plays a role in mRNA transcription coding for enzymes involved in antioxidant reactions [57,58]. With high concentrations of soluble NPs, cells failed to restore this imbalance, leading to oxidative stress, oxidative damage and cytotoxicity. With insoluble NPs like TiO_2_, despite ROS production, no oxidative stress or nuclear translocation of NF-κB were detected. Cells were able to maintain their antioxidant potential even at high concentrations. In the literature a wide range of nanomaterials have been shown to produce ROS and induce oxidative stress, leading to cytotoxicity both *in vivo *and *in vitro *[12,59-62]. Our study hypothesized that ROS production, oxidative stress and cytotoxicity induced by CdS and ZnO NPs are partly linked to the release of Cd^2+ ^and Zn^2+ ^ions, respectively. Cadmium metal indirectly generated free radicals, as shown some years ago. In the cellular environment, Cd^2+ ^ions can replace iron and copper in various cytoplasmic and membrane proteins (e.g. ferritin, apoferritin), thus increasing the amount of unbound free or chelated copper and iron ions, which then participate in oxidative stress via Fenton reactions. Similar results were observed by Li *et al.*, [63], suggesting that CdS QDs have the capacity to generate free radicals indirectly and induce oxidative stress and cytotoxicity by releasing Cd^2+ ^in cells. Complementary studies with their micro-counterparts showed that oxidative stress can also be linked to the reactive surface of the particle, thus explaining differences in cytotoxicity. However, the difference between micro and nano NPs cytotoxicity may also be promoted by an easier solubilization of nano-Cd. Zn is an essential metal for the human organism, and is involved in antioxidant mechanism, activity of transcription factor and protein phosphorylation [42]. Moreover, Zn is a redox inert metal and does not participate in the oxidation-reduction reaction. A number of *in vivo *and *in vitro *studies have also investigated the toxicity of ZnO NPs, which is strongly related to ROS production and oxidative stress induction. This toxicity was directly related to particle dissolution and release of toxic Zn^2+^, but the mechanisms of toxicity remain unclear [52]. Our data showed dissolution of ZnO NPs in media. It was observed that part of the soluble ZnO was sufficient to induce toxicity and the IC_50 _was similar to the value obtained with ZnO NPs. These results confirmed that the origin of the toxicity was strongly linked to the solubility of ZnO NPs. In contrast, studies on TiO_2_, as insoluble particle, suggested that the production of ROS was linked to the reactive surface, which depends on crystalline structure, size and metal composition [50]. Xia *et al. *[52] found similar results where solubility was an important parameter in cell responses.

Further investigations are required to better elucidate the effects of particle solubility on cellular effects and their toxic mechanisms. Oxidative stress acts upon the signaling pathways of MAPK and on transcription factors [64-66]. *In vitro *studies and *in vivo *studies on animal models have provided evidence that upregulation of NF-κB isoform (in tubular epithelial cells, podocytes, mesangial cells, macrophages) has a pathogenic role in mediating chronic inflammation in chronic kidney disease [67,68]. NPs were found to stimulate an increase in cytosolic Ca^2+ ^concentration that may cause activation of NF-κB. Calcium signaling events may also drive further production of reactive oxygen species, leading to a positive feedback mechanism. Further investigations using different inhibitors of NF-κB, such as IκB, or other transcription factors (Nfr-2 and AP-1) are required to elucidate the effects of particles on signaling.

## Conclusion

In this study, we described for the first time the effects of three different inorganic NPs in renal cells *in vitro*. We report their ability to exert different cytotoxic effects. These cytotoxic effects were correlated with metal composition, particle scale and degree of solubility. ROS production and oxidative stress induction in glomerular and tubular cells clearly indicated the nephrotoxic potential of CdS and ZnO NPs. Nephropathies are pathologies in which ROS undoubtedly play a role. Imbalance between ROS and antioxidants results in a destructive effect on mesangial cells by altering lipid metabolism. This phenomenon is frequently observed in patients with glomerulonephritis and nephritic syndrome, and is also a major mechanism in the nephrotoxicity induced by Cd metal. Moreover, cellular uptake of NPs into cytoplasmic vacuoles may lead to an accumulation over a long period, leading to renal injury on long-term exposure. Further investigations on NPs accumulation effects may be necessary. The validity of these *in vitro *results is nevertheless limited and *in vitro *toxicity assays show more severe cell damage than *in vivo *investigations. Nevertheless, *in vitro *measurements are not only rapid and easy to perform, but also could be used for pre-screening NPs to help better predict their *in vivo *toxicity.

## Methods

### Chemical

NPs of TiO_2 _(15 nm) and ZnO (<100 nm) were from Sigma Aldrich (St-Quentin-Fallavier, France). CdS NPs were manufactured by Institute of Chemistry of Condensed Matter Physics of Bordeaux (ICMCB, Bordeaux, France). The CdS NPs were prepared via a colloidal approach in aqueous solution. CdSO_4 _(99.99%) and sodium polyphosphate (80 mesh) were purchased from Aldrich and used as received. A solution of CdSO_4 _(300 μM) and sodium polyphosphate (300 μM) was prepared and introduced in a three-necked flask. The pH was adjusted to 9.5 with NaOH. The solution was degassed by bubbling with Ar(g) for 3 h at 80°C. The solution was then bubbled with mixed H_2_S/Ar for 30 min at 80°C with stirring, at which point it was left under Ar (without heating) for about 3 h to eliminate any excess of H_2_S. This procedure yielded a yellow solution consisting of CdS NPs of 8 nm diameter. For comparison, large-sized TiO_2 _and ZnO particles used were purchased from Sigma Aldrich (St-Quentin-Fallavier, France) and CdS microparticle was from Alfa Aesar (Schiltigheim, France). All products used for cell culture (RPMI and DMEM/F12 media, PBS Phosphate Buffer Saline) were purchased from Lonza (Verviers, Belgium) (exceptions mentioned). WST-1 was from Roche Diagnostics (Meylan, France). MTT, Dichlorodihydrofluorescein diacetate (DCFH-DA), Tert-Butyl hydroperoxide (TBHP), 5-Sulfosalicylic acid, NaH_2_PO_4_, Na_2_EDTA, 2-vinyl pyridine (2VP), N-Acetyl-Cysteine (NAC), Glutathione Reductase, NADPH, 5,5'-dithio-bis(2-nitrobenzoic acid) (DTNB), NP40 and Bovin Serum Albumin (BSA) were from Sigma Aldrich (St-Quentin-Fallavier, France).

### Preparation of particles

Particle stock suspensions of micro and nano TiO_2 _and ZnO particles were prepared (2 mg/ml) in RPMI 1640-serum-free medium without phenol red, L-glutamine and antibiotics. Suspensions were frozen immediately after brief sonication (20s, 9 times) (Vibracell 75186, 130 W, 56-60 Hrz). Additionally, prior to each cell culture experiment, in order to distribute the particles in the working solution as evenly as possible, the samples were processed again by sonication (20s, 3 times). The same particle preparation was used for CdS microparticle. To directly compare compounds, usually expressed as μg/ml or μM, all CdS, ZnO or TiO_2 _concentrations were expressed in μg/cm^2 ^of metal element used.

### Characterization of particles

Characterization of particles was performed in RPMI 1640-serum free medium. Transmission electron microscope (TEM, JEOL 2000FX) was used to determine NPs size, shape and aggregation state. Particles were examined after subsequent deposition onto collodion-coated carbon grids. SIS software for the TEM camera was calibrated to measure NPs size. Turbidimetry measurements were also used to characterize particle dispersion rates. Measurements were carried out in RPMI 1640-serum free medium or in ultrapure deionized water, using a HACH 2100AN turbidimeter which includes a tungsten-filament lamp and 90° and 180° light detectors. Turbidity, expressed in Nephelometric Turbidity Units (NTU), quantifies the degree to which light travelling through a sample is scattered by the suspended particles. Zeta potential, using a Malvern Zetasizer (Nano Series DTS 1060, Malvern Instruments S.A., Worcestershire UK), determines the electrical potential that exists at the shear plane of NPs in solution. Stock solutions were also analyzed for the possible presence of metal impurities using a validated ICP-OES method [69] (IRIS Intrepid XSP II Thermo Electron). TiO_2_, ZnO and CdS NPs solutions (20 μg/ml) were acidified by HNO_3 _for 24 h and analyzed.

### Cell cultures

Human IP15 mesangial cells, a gift from Dr. I. Dubus (University of Rouen, France), were cultured in RPMI 1640 medium containing penicillin (100 U/ml), streptomycin (100 μg/ml) and amphotericin B (0.25 μg/ml), 2 mM L-glutamine, sodium pyruvate, non-essential aminoacids and 10 mM Hepes supplemented with 10% inactivated fetal bovine serum (FBS) (Eurobio, Les Ullis, France). Human Kidney (HK-2) epithelial cells were purchased from the American Type Culture Collection (ATCC, CRL-2190). Cells were grown in DMEM/F12 (Dulbecco's Modified Eagles Medium) supplemented with 2 mM L-glutamine, streptomycin (100 U/ml) and penicillin (100 μg/ml) supplemented with 10% FBS. IP15 and HK-2 cells line present the morphological and functional characteristics of glomerular mesangial cell and proximal tubular cell previously described in L'Azou *et al. *[28] and Gunness *et al.*, [29]. Both cell cultures grew in 75 cm^2 ^plastic culture flasks (Greiner BioOne, Courtaboeuf, France) and were maintained in 5% CO_2 _- 95% air atmosphere. The media were changed every 2 days and cells were trypsinized when necessary (0.05% trypsin - 0.53 mM EDTA).

### Electron Microscope (Transmission EM and Scanning EM) observations and X-Ray analysis

Sub-confluent cells in 60 mm Petri dishes were exposed to NPs for 24 h, washed with PBS and fixed by 2.5% glutaraldehyde in 0.1 M sodium cacodylate buffer at 4°C for 2 h and post-fixed in 1% osmium tetraoxide (pH 7.4). After dehydration in ascending grades of ethanol, cells were subsequently embedded in epoxy resin. Ultrafine sections (70 nm) were performed using an ultra-microtome before observation using a Hitachi H7650 electron microscope. The chemical nature of NPs (Ti, Zn, Cd) observed in the cells was confirmed by energy dispersive X-ray spectrometer (EDS) to measure the wavelength emitted during X spectrum coupled to TEM (JEOL 2000FX). For scanning electron microscopy, cells were grown on glass slides to subconfluence and treated at appropriate concentrations of NPs for 24 h. After exposure, cells were fixed, with PFA 1% and dehydrated to 100% ethanol, cells were dried, sputter coated with gold, and examined in a scanning electron microscope (Hitachi S-2500), operating at 10-15 kV.

### Cell viability assays

Effects of particles on IP15 and HK-2 cells viability were evaluated using three methods: Neutral Red (3-amino-7-dimethylamino2-methylphenazine-hydrochloride), MTT [3-(4,5-dimethylthiazol-2-yl)-2,5diphenyl tetrazolium bromide] and WST-1 [2-(4-iodophenyl)-3-(4-nitrophenyl)-5-(2,4-disulfophenyl)-2H-tetrazolium] assay. Mitochondrial activity was assessed with the MTT and WST-1 assay based on cleavage of the tetrazolium salt to a formazan dye by succinate-tetrazolium reductase, which exists in the mitochondrial respiratory chain and is active only in viable cells. On the other hand, Neutral red is a supravital dye that is taken up by viable cells with intact plasma membrane and stored in lysosomes. Sub-confluent cells in 96-well plates were exposed 24 h to varying concentrations of particles diluted in serum-free medium. In relation to the cell surface dishes used (0.32 cm^2^/well) and the volume distributed (0.1 ml/well), different concentrations ranging from 2 to 512 μg/ml, 0.32 to 64 μg/ml and 20 to 180 μM (for TiO_2_, ZnO, and CdS respectively) were prepared and corresponded from 0.625 to 160, 0.1 to 20 and 0.7 to 6.33 μg/cm^2 ^(for TiO_2_, ZnO, and CdS respectively). For MTT assay [70], after NPS exposure and additional 3 h incubation with culture medium containing MTT (0.5 mg/ml), formazan salts were solubilized with DMSO. For WST-1 assay, the reagent was added directly to culture medium without additional solubilization step. In the Neutral Red assay, after exposure, cells were washed with NaCl solution (9 mg/ml) and incubated at 37°c for 3 h in culture medium containing the 4% Neutral Red. The cells were washed and fixed with formaldehyde-CaCl_2 _solution, and then extracted by adding acetic acid-ethanol solution. Absorbance was measured at 570 nm, 450 nm and 540 nm compared to a 630 nm reference using a multiscan photometer (Titertek Plus II) for MTT, WST-1 and NR assay, respectively. Data from at least 3 independent triplicates were expressed as percentage of dead cells compared to a control from the same experiment.

### Measurement of ROS production

The intracellular reactive oxygen species (ROS) was determined in serum-free media using 2-7' dichlorodihydrofluorescein diacetate (DCFH-DA). DCFH-DA is a stable, non-fluorescent molecule that is hydrolyzed by intracellular esterases to non-fluorescent 2-7'-dichlorodihydrofluorescein (DCFH), which is rapidly oxidized in the presence of hydroxyl radical to a highly fluorescent compound (DCF) [71]. Sub-confluent cells grown in 60 mm Petri dishes were incubated 15 min with 10 or 20 μM of DCFH-DA for IP15 and HK-2, respectively. Cells were washed with PBS and treated with different concentrations of particles for 4 h. Tert-Butyl hydroperoxide (TBHP) solution served as a positive control for the induction of ROS in cells. After exposure, cells were scraped off, lysed by sonication and centrifuged. Supernatants were collected and ROS levels were determined at excitation 480 nm and emission 520 nm wavelengths using a fluorimeter (Kontrol Instrument, SFM 25, Eching, Germany). Data from at least 3 independent triplicates are reported as fluorescence intensity percentage and expressed as mean fluorescence ratio (fluorescence of exposed cells/fluorescence of unexposed control from the same experiment).

### Measurement of oxidative stress

Intracellular reduced (GSH) and oxidized (GSSG) glutathione levels were measured according to the method of Tiezte [72]. Cells in 60-mm Petri dishes were incubated with different concentrations of NPs for 24 h. Tert-Butyl hydroperoxide solution was used as control to induce oxidative stress by ROS production. N-Acetyl-Cysteine (NAC), a precursor of glutathione, was used as control for increasing intracellular glutathione level. After several washing steps with PBS, cells were centrifuged, resuspended in PBS and lysed by sonication. Aliquot of cell suspension was taken for protein quantification using BioRad protein assay based on the Bradford method [73]. The remaining cell suspension was mixed with 5% sulfosalicylic acid solution and protein precipitant was removed after centrifugation (10 min at 4°C). For quantitative determination of total GSH (tGSH) level, 25 μL of the supernatant was mixed with DTNB mixture [100 μM NaH_2_PO_4_, 10 μM Na_2_EDTA], 1 mM DTNB, 1 mM NADPH and 50 U/mL GSH reductase (GR). tGSH level was measured photometrically at 405 nm. For measurement of the GSSG level, the thiol-scavenging reagent 2-vinylpyridinium (2-VP) was used to prevent the participation of GSH in the enzymatic assay. Therefore, supernatant and 2-VP were mixed and left for 1 h at 26°C before addition of DTNB. The GSSG level was determined photometrically at 405 nm. All glutathione (GSH, GSSG) levels were determined as nmol/mg protein and results are given as percent relative to control cells.

### Immunostaining evidence on NF-κB nuclear translocation

After NPs exposure, cells growing on coverslips were fixed with 4% para-formaldehyde for 30 min at 4°C, washed with PBS, and permealized with 0.1% NP40 at 4°C. The cells were again washed with PBS, blocked with 2% BSA, 0.1% glycine for 1 h. Cells were treated with diluted (1/60) anti-NF-κB p56 subunit antibody (Santa Cruz) for 2 h at room temperature. After additional washing steps, cells were incubated with FITC-conjugated anti-rabbit IgG, diluted 1/200 (Santa Cruz) for 1 h at room temperature, washed with PBS and then stained with DAPI for 10 min at room temperature. The cells were washed twice more with PBS and were mounted before viewing under fluorescent microscope (Nikon Eclipse 80i). Images were captured using digital camera (DXM 1200c) controlled by NIS-element software. NPs effects were expressed as percentage of cells with high nuclear intensity compared to untreated cells.

### Statistics

NP size (mean ± SD) obtained by TEM using ImageJ software were calculated by measuring over 100 NPs in random fields of view. For cytotoxicity experiments, results were calculated using the formula (100 - (Absorbance treated sample × 100/Absorbance control sample)) and expressed as mean ± SE of at least three triplicate independent experiments. Non-linear Boltzman regression analysis was performed using the Origin^® ^software (Origin Lab. Corp, Northampton, USA) and the IC_50 _(defined as concentration which induces 50% cell mortality increase) were calculated. For ROS and glutathione measurements, statistical analysis was performed for the experiments conducted in at least triplicate using Student's *t*-test. For all experiments, results with *p*>0.05 were considered to be statistically significant.

## Competing interests

The authors declare that they have no competing interests.

## Authors' contributions

IP participated in the design of the study and contributed to all the sections; IP carried out the cytotoxicity and the ROS studies; BB prepared cell specimens for electron microscopy and performed SEM observations; MT helped to prepare and characterize CdS NPs; ED performed CdS NPs; COC performed the ICP/OES assays and helped to draft the manuscript; BL designed the study, contributed to the cytotoxicity study and drafted the manuscript. All authors read and approved the final manuscript.

## Supplementary Material

Additional file 1 **Soluble fraction of metal in the supernatant was determined by ICP-OES**. ZnO or CdS NPs were added at different concentrations in RPMI 1640 serum-free medium for 24 h at 37°C. After centrifugation, concentrations of Zn^2+ ^or Cd^2+ ^in supernatant were analyzed by ICP-OES. Data (n > 3) were expressed as soluble fraction metal ([Cd2+] or [Zn2+]) μg/ml. Click here for file

Additional file 2 **Effects of ZnO and CdS NPs on the mortality of (a) IP15 and (b) HK-2 cells, determined using Neutral Red and MTT cytotoxicity assays**. Cells were exposed in RPMI serum-free medium with different concentrations of NPs for 24 h. Results are expressed as the percent of cell mortality compared to the control. Click here for file
