# Supplementary figures and images for: Cytotoxicity and oxidative stress induced by different metallic nanoparticles on human kidney cells
Source: Part Fibre Toxicol. 2011 Mar 3;8:10. doi: 10.1186/1743-8977-8-10 (PMC3058043; doi:10.1186/1743-8977-8-10)

## Slide 1
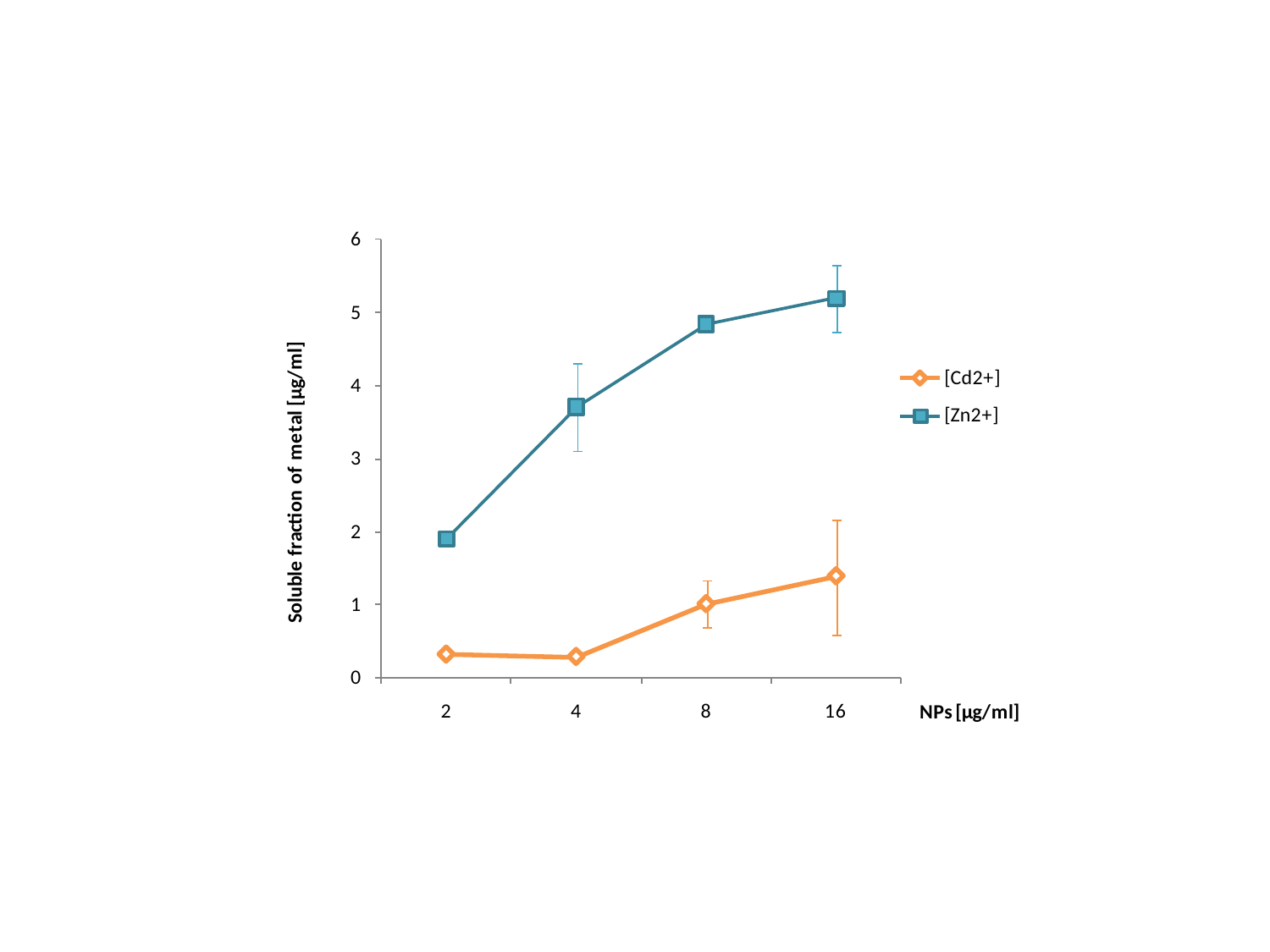

Supplement: Additional file 1 — Soluble fraction of metal in the supernatant was determined by ICP-OES. ZnO or CdS NPs were added at different concentrations in RPMI 1640 serum-free medium for 24 h at 37°C. After centrifugation, concentrations of Zn2+ or Cd2+ in supernatant were analyzed by ICP-OES. Data (n > 3) were expressed as soluble fraction metal ([Cd2+] or [Zn2+]) μg/ml. [file 1743-8977-8-10-S1.PPT]

## Slide 1
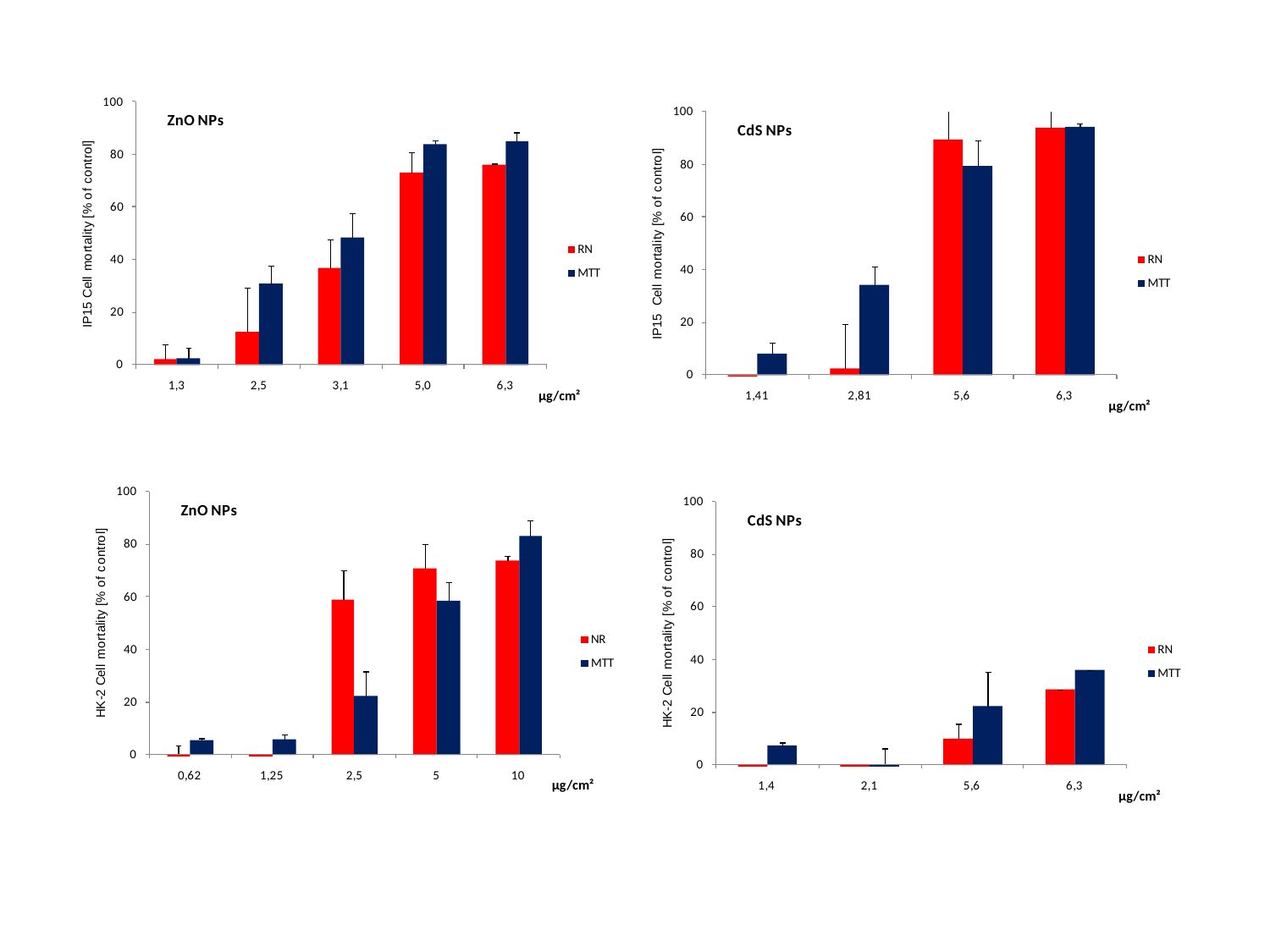

Supplement: Additional file 2 — Effects of ZnO and CdS NPs on the mortality of (a) IP15 and (b) HK-2 cells, determined using Neutral Red and MTT cytotoxicity assays. Cells were exposed in RPMI serum-free medium with different concentrations of NPs for 24 h. Results are expressed as the percent of cell mortality compared to the control. [file 1743-8977-8-10-S2.PPT]
